# Survey on technical management of strawberries in Morocco and evaluation of their post-harvest microbial load

**DOI:** 10.3389/fmicb.2022.1115340

**Published:** 2023-01-10

**Authors:** Abir El-araby, Amal Azzouzi, Iman Msegued Ayam, Khaoula Filali Samouh, Faouzi Errachidi

**Affiliations:** ^1^Functional Ecology and Environment Engineering Laboratory, Faculty of Science and Technology, Sidi Mohamed Ben Abdellah University, Fez, Morocco; ^2^Department of Biology, Immunology and Biodiversity Laboratory, Faculty of Sciences Ain Chock, University Hassan II, Casablanca, Morocco

**Keywords:** climatic conditions, microbiological study, Morocco, post-harvest, pre-harvest, strawberries

## Abstract

The climatic conditions of the growing regions influence the fruit’s microbiological quality and their tolerance to post-harvest pathogens. The present work aims to identify the prevalence of bacterial and fungal strains of strawberries (Fragaria **×** ananassa) in the Gharb and Loukkos regions of Morocco. Thus, to establish a correlation between the microbial load and the climatic conditions of the two targeted regions. The bacteriological analyses were studied according to the International Organization for Standardization (ISO) methodologies. Regarding the mycological study, fungal species determination was performed using identification keys. Yeast species determination was done using genus analysis, assimilation, and fermentation tests. Emberger bioclimatic quotients (Q_2_) were calculated for the Gharb and Loukkos regions and bioclimatic stages were determined. *Salmonella* spp. was not detected in the studied samples. However, *Listeria monocytogenes* and *Escherichia coli* were isolated from the Gharb samples. Sulfite-reducing clostridia spores were found in two Gharb samples versus one Loukkos sample. Coagulase-positive staphylococci were negative in all samples analyzed. Loukkos and Gharb regions were contaminated by *Bacillus cereus* with percentages of occurrence of 5.2 and 7.8%, respectively. The fungi found in strawberries from both regions were *Aspergillus niger*, *Botrytis cinerea*, *Fusarium* spp., *Penicillium* spp., *Rhizopus* spp., and *Alternaria alternata* with a significant predominance in the Loukkos samples. Indeed, a marked presence is noted for *Candida sake* and *Rhodotorula glutinis* in strawberries from Loukkos. Gharb is located on the semi-arid stage, while Loukkos is located on the sub-humid stage. Climatic conditions have a strong influence on plant microbial load, which explains the prevalence of bacteria in strawberries from Gharb and the prevalence of fungi in strawberries from Loukkos.

## 1. Introduction

Fruits are living organisms with a shelf life largely influenced by ambient temperature, relative humidity, precipitation, atmosphere composition during and after harvest, and type and degree of infection by microorganisms (Singh and Sharma, 2018). Strawberry fruits are considered to be a source of vitamins, minerals, and natural substances necessary for the proper functioning of the human body. These fruits are considered beneficial to health due to their antioxidant and antibacterial properties (Steinka and Kukułowicz, 2018). In general, red fruits have limited storage potential due to their extreme tenderness, vulnerability to mechanical damage, high respiration and transpiration rates, and susceptibility to fungal spoilage (Matar et al., 2020). These soft fruits are highly perishable, which leads to a rapid deterioration of their appearance and texture once they reach the market, generating significant economic losses for the strawberry industry worldwide.

Therefore, it is essential to apply adequate practices before and after harvesting strawberry fruits. The most widely used method to maintain strawberry quality is rapid post-harvest cooling also continuous low-temperature (0–4°C) storage (Tahir et al., 2018). High CO_2_ atmospheres are one of the best methods to control post-harvest strawberries spoilage (Nakata and Izumi, 2020). Other postharvest techniques have also been applied to extend the shelf life of these susceptible fruits, such as gamma rays (de Jesus Filho et al., 2018), chemical application (Shu et al., 2020), and edible coatings (Mohammadi et al., 2021). Post-harvest treatments of strawberry fruits are of great importance to improve the quality and shelf life of these fruits considered extremely perishable, while minimizing losses and maximizing benefits.

Rots development caused by certain fungal pathogens is one of the major causes of postharvest losses in strawberries. Fungal strains commonly affecting strawberries in postharvest are *Botrytis cinerea*, *Rhizopus stolonifer*, *Penicillium* spp., and *Aspergillus* spp. (Feliziani and Romanazzi, 2016; Saleh and Al-Thani, 2019; Weber and Hahn, 2019; Trinetta et al., 2020). Studies have reported that microflora isolated from strawberries included bacteria, such as *Escherichia coli*, *Bacillus cereus*, *Staphylococcus aureus*, and *Staphylococcus epidermidis* (Yoon et al., 2010; Delbeke et al., 2015; Oliveira et al., 2019). Data from the literature confirm the probable presence of microorganisms such as *Salmonella* spp., *Listeria monocytogenes*, and *B. cereus* on strawberry fruits surface (Dziedzinska et al., 2018; Ortiz-Solà et al., 2020). In fact, the climatic conditions of the locality of origin have a strong influence on the microbial load of fruits and vegetables. Pre-harvest weather factors, including rainfall, humidity, and temperature, affect the microbiological quality of the fruit and its tolerance to infection and the development of post-harvest pathogens (Machado-Moreira et al., 2019; Bui et al., 2021).

The present work aims to identify the prevalence of bacterial and fungal strains of strawberry fruits (Fragaria **×** Ananassa) in the Gharb (Rabat-Sale-Kenitra) and Loukkos (Tangier-Tetouan-Al Hoceima) regions. Thus, to establish a correlation between microbial load and climatic conditions of the two targeted regions.

## 2. Materials and methods

### 2.1. Strawberry sampling

**Figure 1:**
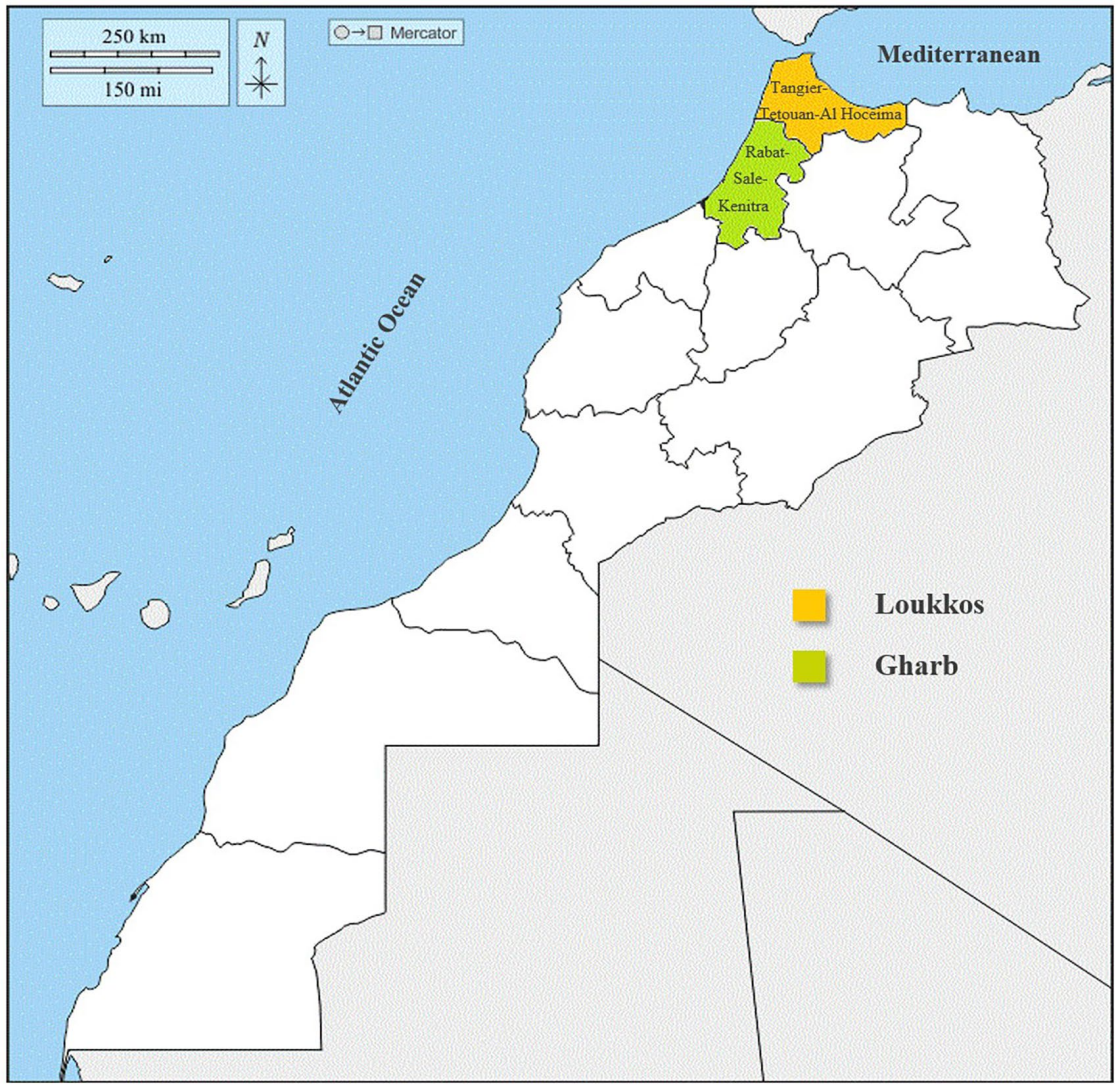
Sampling map of strawberries in the Loukkos and Gharb regions (Morocco).

In this microbiological study, we focused mainly on the two areas closest to our city (Fez) to avoid strawberry samples quality deterioration. Strawberry fruit (Fragaria **×** Ananassa) samples were collected from two suppliers during the year 2020. The samples (*n* = 50) were collected from two sites (one located in the Gharb area and the other located in the Loukkos area) and stored at 4°C at the Laboratory of Functional Ecology and Environment Engineering on the same days (Figure 1).

### 2.2. Bacteriological study of strawberry samples from the Loukkos and Gharb regions

#### 2.2.1. Samples preparation

Twenty-five grams (25 g) of each strawberry fruit sample were suspended in 225 ml of sterile buffered peptone water (BPW) solution and homogenized with a grinder (Stomacher^®^ 400 circulator) for 2 min. Dilution series were prepared from stock solution. A volume of 0.1 ml of each dilution was transferred to Petri dishes surface containing culture media suitable for each target microorganism. However, for the detection of *L. monocytogenes*, 25 g of each sample were homogenized in 225 ml of PALCAM broth and incubated at 30°C for 24 h.

#### 2.2.2. Bacteriological analysis

Microbiological analyses were studied according to the International Organization for Standardization (ISO) methodologies. The bacteriological food safety and quality parameters studied were: *E. coli* (ISO, 2001), *Salmonella* spp. (ISO, 2002), *L. monocytogenes* (ISO, 1996; ISO, 1998), *B. cereus* (ISO, 2004), Coagulase-positive staphylococci (ISO, 1999), and Sulfite-reducing clostridia spores (IPQ, 1986).

Presumptive *L. monocytogenes* colonies on PALCAM agar were streaked on sheep blood agar to assess hemolysis, carbohydrate zymogram (rhamnose, xylose, and mannitol), and the Christie-Atkins-Munch-Petersen (CAMP) test (Ortiz-Solà et al., 2020). For suspected *Salmonella* spp. colonies, biochemical confirmation was performed with Triple Sugar Iron (TSI) agar, urea agar, and L-lysine decarboxylase medium (Uyttendaele, 2014). For the determination of *E. coli* sample was placed in Petri dish center, subsequently, 20 ml of violet red bile agar (BD Difco, heated to 45°C) was added. After solidification, agar was overlaid with 5 ml of violet red bile agar (double strength) supplemented with 4-Methylumbelliferyl-β-D-glucuronide. The inverted plates were incubated at 35°C for 24 h, and colonies fluoresced under UV light as *E. coli* bacteria (Cárdenas et al., 2013).

For detection of Sulfite-reducing clostridia spores, 10 ml of each sample in buffered peptone water (BPW) solution was heat-shocked at 80–85°C for 10 min before culture to inactivate vegetative bacteria and enhance sporulation phenomenon. Samples were then transferred to 10 ml of differential reinforced clostridium medium (DRCM) and incubated under anaerobic conditions in a jar at 37°C for 5 days. This method is based on ferric Sulfite reduction to iron sulfide and samples are considered positive when color changes (black dots) (Oliveira et al., 2019). Bacilli showing typical *B. cereus* group growth, i.e., rough, dry colonies with a pink-purple background surrounding an egg yolk precipitation, were counted, isolated, and selected for hemolytic activity, tested on sheep blood agar plates after 1 day of incubation at 30°C. Convex black colonies of staphylococci, with or without halo on Baird-Parker medium for 24–48 h at 37°C, were isolated and tested for catalase reaction and coagulase activity using rabbit plasma (Evêncio-Luz et al., 2012).

### 2.3. Mycological study of strawberry samples from the Loukkos and Gharb regions

#### 2.3.1. Molds study

Analysis of mycoflora associated with strawberry fruits was performed using the modified Buvard method (Benkirane, 1995). Strawberries with lesions were disinfected with 1% sodium hypochlorite. Then, strawberries pieces were placed in Petri dishes containing Potato Sucrose Agar (PSA) and incubated for 7 days in the dark at 24°C. Optical microscopic examination of the different cultures allowed for the determination of fungal species using the keys identification of Gilman (1957), Tarr (1962), Ellis (1971), Chidambaram et al. (1974), Domsch et al. (1980), and Champion (1997). Contamination percentage by different fungal species is calculated according to Ponchet (1966) method which defines the isolation frequency of the different fungi from 100 lesions present on the studied fruits according to the following formula (Eq. 1):


(1) $$PC\;\left(\%\right)=\frac{NFI}{NTF}\times 100$$

- PC is the percentage of contamination
- NFI is the number of lesions infected with a given fungal species
- NTF is the total number of lesions.

#### 2.3.2. Yeasts study

Yeasts were isolated on YPG agar medium (Y: 1% yeast extract, P: 2% peptone, and G: 2% glucose). Three times a week for 5 weeks in June and July 2020, plastic trays each containing 500 g of strawberries were harvested during the harvest period. Each tray was placed in a sterile plastic bag at the farm and sampled at the laboratory 3 h later. Whole strawberries with calyxes (100 g) were mixed with 100 ml sterile chilled KH_2_PO_4_ (0.1 M) for 45 s at half speed, then 45 s at full speed. Four successive dilutions of 10 times were performed aseptically with KH_2_PO_4_ (0.1 M). An aliquot of 100 μl was put on Petri plates containing YPG agar supplemented with two antibiotics chloramphenicol and kanamycin at 5 μg/ml and incubated at 30°C for 2 days.

Yeast colonies were counted on all Petri dishes and at all dilution levels when possible, after the longest possible incubation before colonies coalesced. Counts were expressed as the number of yeast per g of strawberries. Yeast purification was performed by at least five successive streaking on YPG agar (pH 4) incubated at 30°C. The identification was done on Wikierham medium for the assimilation (Auxanogram) and fermentation (Zymogram) tests. The identification key is the one proposed by Kreger-van Rij (1984).

### 2.4. Bioclimatic study of the two regions Loukkos and Gharb

A climatic study (Emberger bioclimatic quotient) was carried out to know the climate effect on strawberry fruits contamination in the two targeted regions (Gharb and Loukkos), the Emberger bioclimatic quotient (Q_2_) was calculated according to the following formulas (Table 1). Climatograms were developed to identify the bioclimatic stage of the two stations studied (Loukkos and Gharb).

**Table 1:** Emberger bioclimatic quotient (Q_2_) formulas.

Formulas	References	Climatological parameters
Q_2_ = 2000*P/ (M + m + 546.4)*(M-m) Q_2_ = 3.43*P/ (M-m)	Mokhtari et al. (2013) Stewart (1968)	P: Annual precipitation in mm/m^2^/year M: Maximum temperature of the warmest month in °C m: Minimum temperature of the coldest month in °C
Q_2_ = 2000*P/(M^2^−m^2^)	Emberger (1942)	P: Annual precipitation in mm/m^2^/year M: Maximum temperature of the warmest month in °K m: Minimum temperature of the coldest month in °K

### 2.5. Statistical analyses

The statistical analyses were developed with SPSS statistical software. All data were analyzed for significant differences by applying analysis of variance test (ANOVA), with a probability of *p* ≤ 0.05. Results of Coagulase-negative staphylococci and presumptive *B. cereus* strains were expressed as a percentage of positive samples.

## 3. Results and discussion

### 3.1. Bacteriological study of strawberry samples from the Loukkos and Gharb regions

Strawberry samples microbiological quality was determined by the detection of *Salmonella* spp., *L. monocytogenes*, *E. coli*, and Sulfite-reducing clostridia spores and by enumeration of *B. cereus* and Coagulase-positive staphylococci according to the International Organization for Standardization (ISO). The occurrence of *Salmonella* spp., *L. monocytogenes*, *E. coli*, and Sulfite-reducing clostridia spores is presented in Table 2.

**Table 2:** Occurrence of *Salmonella* spp., *Listeria monocytogenes*, *Escherichia coli*, and Sulfite-reducing clostridia spores in strawberry samples.

Samples source	*N*	*Salmonella* spp.	*L. monocytogenes*	*E. coli*	S. R. clostridia
Loukkos	25	ND	ND	ND	1
Gharb	25	ND	1	1	2

N: Number of analyzed samples. ND, not detected.

*Salmonella* spp. was not detected in all samples from the two studied regions (*n* = 50). However, *L. monocytogenes* and *E. coli* were isolated from only two fruit samples of the Gharb region. Sulfite-reducing clostridia spores were found in two samples tested from the Gharb locality against one sample from the Loukkos area. Samples from the Gharb region have a relatively high prevalence of these microorganisms with 4 positive samples in total. Bacterial contamination level was below 100 CFU/g in both targeted areas (Gharb and Loukkos).

Table 3 shows the prevalence results of *B. cereus* and Coagulase-negative staphylococci in strawberry samples from the two targeted regions (Loukkos and Gharb).

**Table 3:** Prevalence (%) of presumptive *Bacillus cereus* and coagulase-negative staphylococci in strawberry fruit samples.

Samples source	*N*	Percentage of contaminated samples	
		Coagulase-negative staphylococci	Presumptive *Bacillus cereus*
Loukkos	25	1.6%	5.2%
Gharb	25	2.4%	7.8%

N: Number of analyzed samples.

Coagulase-positive staphylococci were negative in all samples from the two studied regions (*n* = 50). Therefore, the prevalence of Coagulase-negative staphylococci was reported. Coagulase-negative staphylococci were detected in 1.6 and 2.4% of strawberry samples from Loukkos and Gharb, respectively. According to Table 3, the two targeted areas samples were contaminated by *B. cereus* with occurrence percentages of 5.2 and 7.8% in Loukkos and Gharb, respectively. Samples from the Gharb region have a relatively high prevalence of *B. cereus* and Coagulase-negative staphylococci compared to those from the Loukkos region.

In this study, all samples from the Loukkos region tested negative for *Salmonella* spp., *L. monocytogenes*, and *E. coli*. However, two strawberry samples from the Gharb region were contaminated with *L. monocytogenes* and *E. coli*. Sulfite-reducing clostridia spores were detected in both targeted areas, with a relatively high prevalence for the Gharb region. Coagulase-negative staphylococci and *B. cereus* were isolated from samples collected in the Gharb and Loukkos localities, samples from the Gharb region being the fruits with the highest occurrence percentage of these microorganisms. The overall results of this bacteriological analysis indicated that the bacteriological quality of the strawberry samples tested was satisfactory.

A study done by Ortiz-Solà et al. (2020) in Spain, reported that all tested samples (fresh and frozen strawberries) were negative for *Salmonella* spp., *L. monocytogenes*, and *E. coli.* These results are in accordance with our bacteriological analyses of strawberry samples from the Loukkos region. A survey conducted by Delbeke et al. (2015) in Belgium, reported that no *Salmonella* spp. or Shigatoxin-producing *E. coli* were isolated from the analyzed strawberry samples. In the same report, generic *E. coli* was present in only two of 72 strawberry samples at levels of 1.0 and 3.0 log CFU/g. Similarly, Macori et al. (2018) evaluated freshly harvested berries (Raspberries, blueberries, blackberries, and currants) and did not find any *Salmonella* spp. and Shigatoxin-producing *E. coli* in the tested samples.

Results were found by Dziedzinska et al. (2018) in Czech Republic, where detection of *L. monocytogenes* was reported as sporadic with only one positive sample among all field strawberries (0.6%) and one positive sample of purchased strawberries (1.4%). In another study, Delbeke et al. (2015) reported that *L. monocytogenes* was found in 3.8% of strawberry samples. Sulfite-reducing clostridia species are ubiquitously distributed in the environment and can be easily isolated from vegetation (Palese et al., 2009). This explains the presence of these microorganisms in two strawberry samples from the Gharb locality and one sample from the loukkos area. Oliveira et al. (2019) reported the presence of sulfite-reducing clostridia spores in berry fruits. However, blackberries had the highest prevalence of these microorganisms with 35 positive samples in total.

A study performed by Graça et al. (2017) in southern Portugal, reported the absence of coagulase-positive staphylococci in fresh-cut strawberries. Similar results were found by Oliveira et al. (2019) in berries. However, *Staphylococcus* spp. was detected in 10.1, 11.5, 44.9, and 14.4% of strawberry, blueberry, raspberry, and blackberry samples, respectively. *B. cereus* is ubiquitous and can be found in a wide range of foodstuffs, soil, raw materials, raw fruits and vegetables, raw herbs, dry foods, and processed foods (Park et al., 2018; Tango et al., 2018). In our study, *B. cereus* was detected in samples from the two targeted localities (Loukkos and Gharb). Kim et al. (2014) reported that *B. cereus* was found in 98 strawberry samples (31.1%) out of 315 total samples analyzed in South Korea.

### 3.2. Mycological study of strawberry samples from the Loukkos and Gharb regions

#### 3.2.1. Molds study

Optical microscopic examination of the different cultures allowed us to determine the fungal species using the identification keys and consultation of the fungal collections of the National School of Agriculture (ENA-Meknes). Table 4 groups the results obtained in the two localities targeted (Loukkos and Gharb) by this microbiological study.

**Table 4:** Prevalence of mold strains contaminating strawberry fruits in the two targeted localities Loukkos and Gharb.

Molds species	Loukkos region	Gharb region
*Alternaria alternata*	2	1
*Aspergillus nidulans*	0	1
*Aspergillus niger*	6	2
*Botrytis cinerea*	5	2
*Colletotrichum acutatum*	1	0
*Fusarium oxysporum*	0	1
*Fusarium* spp.	3	1
*Mucor* spp.	2	0
*Penicillium notatum*	1	0
*Penicillium* spp.	3	1
*Rhizopus* spp.	3	2

From these preliminary results, we notice that *Aspergillus niger*, *B. cinerea*, *Fusarium* spp., *Penicillium* spp., *Rhizopus* spp., and *Alternaria alternata* species are dominant in both study areas with a priority of fungal contamination in strawberry samples from Loukkos locality. Indeed, a marked presence is noted for *A. niger* on strawberry samples, with six positive samples from the Loukkos region and three positive samples from the Gharb region. *B. cinerea* was also found on five samples from Loukkos and two samples from Gharb. Three samples from the Loukkos locality were contaminated with *Fusarium* spp. while only one strawberry sample from the Gharb locality was positive. The same results were reported for *Penicillium* spp. Moreover, the presence of *Rhizopus* spp. was revealed on three samples from the Loukkos region against two samples from the Gharb region. Two strawberry samples from Loukkos and one sample from Gharb were positive for the *Alternaria alternate* strain. However, *Colletotrichum acutatum*, *Mucor* spp., and *Penicillium notatum* were isolated only from the Loukkos samples. While *Aspergillus nidulans* and *Fusarium oxysporum* species were found in the samples from the Gharb region. In general, strawberry samples from the Loukkos area have a relatively high prevalence of these microorganisms with 26 positive samples in total against 11 positive samples in the Gharb area.

According to the molds study, the most common fungi found in strawberry samples from both regions (Loukkos and Gharb) were *A. niger*, *B. cinerea*, *Fusarium* spp., *Penicillium* spp., *Rhizopus* spp., and *A. alternata* with a significant predominance in samples from the Loukkos region. In a study done by Saleh and Al-Thani (2019), samples of cucumber, tomato, strawberry, and orange were tested to identify spoilage agents. Various fungal types were isolated from strawberry samples, including 5 *Aspergillus* spp., 8 *Rhizopus* spp., 3 *Penicillium* spp., and 5 *Botrytis* spp. In the same report, results indicate that strawberry samples contain the greatest fungi variety of all the fruits. *B. cinerea* causes gray mold, the most important disease of strawberries harvested worldwide (Petrasch et al., 2019; Weber and Hahn, 2019). *A. niger* is by far the most common Aspergillus species responsible for the post-harvest rot of fresh fruit, including apples, pears, peaches, citrus fruits, grapes, and strawberries (Pitt and Hocking, 2009).

Strawberries are fruits closer to the ground and therefore are more susceptible to soft rot mainly *Rhizopus* spp. and *Mucor* spp. (Feliziani and Romanazzi, 2016). In a study conducted by Agyare et al. (2020) in the United Kingdom, *Mucor piriformis* and *Rhizopus* spp. were isolated from decaying strawberry fruit. Tournas and Katsoudas (2005) revealed the presence of *Penicillium* spp. in all types of berries tested (strawberries, blackberries, and raspberries). *Penicillium expansum* and some other species of the genus *Penicillium* can cause significant post-harvest losses in strawberries (Feliziani and Romanazzi, 2016). A study carried out by Mouden et al. (2013) marked the presence of *B. cinerea* on strawberry samples, with a percentage of contamination ranging from 48.4 to 58.1%. In the same report, an attack by *A. alternata* was revealed on the samples analyzed with a percentage of contamination ranging from 19.8 to 23.4% depending on the varieties studied. A study done by El-araby et al. (2022) reported that the fungal strain *A. niger* was isolated from spoiled strawberries.

#### 3.2.2. Yeasts study

The analysis of the genus (nitrate assimilation, mycelium form, and sporulation) and the assimilation (Auxanogram) and fermentation (Zymogram) tests of the yeasts (Tables 5, 6) allowed us to establish Table 7.

**Table 5:** Auxanogram of yeast strains contaminating strawberry fruits from the two localities Loukkos and Gharb.

Assimilation tests	Yeast strains				
	*C. sake*	*Cr. albidus*	*G. candidum*	*P. guilliermondii*	*R. glutinis*
Glucose	+	+	+	+	+
Inulin	−	−	−	+	−
Sucrose	+	+	−	+	−
Raffinose	−	+	−	+	−
Melibiose	−	−	−	+	−
Galactose	+	−	−	+	−
Lactose	−	−	−	−	−
Trehalose	+	+	−	+	−
Maltose	−	+	−	+	−
Melezitose	−	+	−	+	−
Methyl α-D-glucoside	−	−	−	+	−
Soluble starch	−	+	−	−	−
Cellobiose	−	+	−	+	−
Salicin	−	+	−	+	−
L-sorbose	−	−	−	−	−
L-rhamnose	−	−	−	−	−
D-xylose	−	+	+	+	−
L-arabinose	−	+	−	+	−
D-arabinose	−	+	−	+	−
D-ribose	−	−	−	+	−
Ethanol	−	+	+	+	−
Glycerol	−	−	+	+	+
Erythritol	−	−	−	−	−
Ribitol	−	−	−	+	+
Galactitol	−	−	−	−	−
D-mannitol	−	+	−	+	+
D-glucitol	−	+	−	+	−
Myo-inositol	−	+	−	−	−
DL-lactate	−	+	−	+	+
Succinate	+	+	−	+	−
Citrate	−	+	−	−	−
D-gluconate	−	+	−	−	−
D-glucosamine	−	−	−	+	−
Nitrate	−	+	−	−	−
Vitamin-free	−	−	−	−	−

**Table 6:** Zymogram of yeast strains contaminating strawberry fruits from the two localities Loukkos and Gharb.

Fermentation tests	Yeast strains				
	*C. sake*	*Cr. albidus*	*G. candidum*	*P. guilliermondii*	*R. glutinis*
Glucose	+−	−	+	+−	−
Galactose	+−	−	−	−	−
Sucrose	−	−	−	−	−
Maltose	−	−	−	−	−
Lactose	−	−	−	−	−
Raffinose	−	−	−	−	−
Trehalose	−	−	−	−	−

**Table 7:** Prevalence of yeast strains contaminating strawberry fruits from the two localities Loukkos and Gharb.

Yeasts species	Loukkos region	Gharb region
*Candida sake*	4	1
*Cryptococcus albidus*	1	0
*Geotrichum candidum*	0	1
*Pichia guilliermondii*	1	1
*Rhodotorula glutinis*	3	0

In our study, the prevalence of yeast strains contaminating strawberry fruits from the Loukkos and Gharb localities was negligible, with a priority on fungal contamination for strawberries from the loukkos. Indeed, a marked presence is noted for *Candida sake* in strawberry samples, with four positive samples from the Loukkos region and one positive sample from the Gharb region. While *Rhodotorula glutinis* was found in only three samples from the Loukkos.

A study by Tournas and Katsoudas (2005) reported the presence of yeasts in only about 5% of raspberries and 3% of strawberry samples. The lower incidence of yeasts may be partly explained by the fact that these organisms cannot penetrate the epidermis of the fruit and infect the internal tissues. In addition, yeasts are probably more sensitive to chlorine and are therefore inactivated or washed out during the disinfection step. Isolation and identification of *Rhodotorula* spp. and *Cryptococcus laurentii* from strawberries could be considered in light of bio-control of spoilage molds (*Rhizopus* spp. and *B. cinerea*; Campaniello et al., 2008). Similar results were reported by Zhang et al. (2007) for *Cryptococcus laurentii* against *Rhizopus* spp. in strawberry fruit.

A study performed by Zhang et al. (2007) reported that *R. glutinis* was isolated from the surfaces of strawberries harvested in unsprayed orchards. Another report mentioned the presence of *R. glutinis* and *Debaryomyces melissophilus* in packaged strawberries of the Belgian variety Elsanta (Ragaert et al., 2006). The most frequently isolated yeasts on strawberries from conventional and organic growers belonged to the genus Candida, Cryptococcus, and Rhodotorula (Jensen et al., 2013). *Geotrichum candidum* was isolated from diseased strawberries by Ma et al. (2018).

### 3.3. Bioclimatic study of the two regions Loukkos and Gharb

#### 3.3.1. Average temperature and precipitation

In Gharb, summers are hot, arid, and generally clear and winters are cool and partly cloudy. Over the course of the year, the temperature generally varies between 8°C and 32°C and is rarely below 4°C or above 37°C. The very hot season in Gharb lasts for 3 months, from June 20th to September 20th, with an average daily maximum temperature above 29°C. The hottest month of the year in Gharb is August, with an average maximum temperature of 31°C and a minimum temperature of 20°C. The cool season in Gharb region lasts 3.5 months, from November 24th to March 7th, with an average daily maximum temperature below 20°C. The coldest month of the year in Gharb is January, with an average minimum temperature of 8°C and a maximum of 18°C.

In Loukkos, summers are hot, humid, and generally clear and winters are long, cool, wet, windy and partly cloudy. During the year, the temperature generally varies between 9°C and 29°C and it is rarely below 5°C or above 36°C. The very hot season in Loukkos region lasts 3 months, from June 22nd to September 22nd, with an average daily maximum temperature of over 27°C. The hottest month of the year in Loukkos is August, with an average maximum temperature of 29°C and a minimum of 20°C. The cool season in Loukkos lasts 3.8 months, from November 25 to March 17, with an average daily maximum temperature of <19°C. The coldest month of the year in Loukkos is January, with an average minimum temperature of 9°C and a maximum of 17°C.

**Figure 2:**
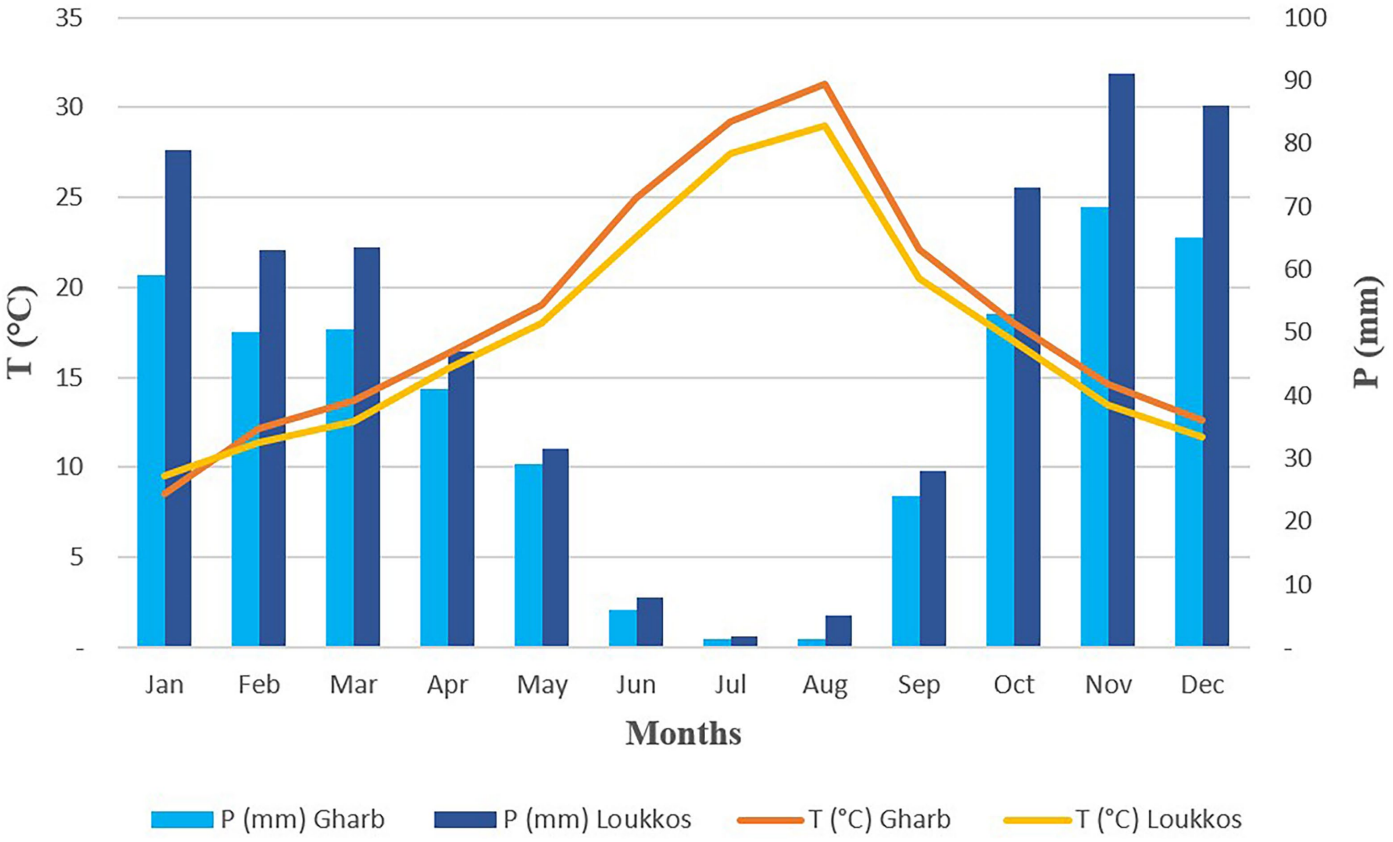
Umbrothermal diagram (temperature and precipitation) of the two regions Gharb and Loukkos.

In the Gharb region, the rainy period of the year lasts 8 months, from September 20th to May 21st, with rainfall of at least 13 mm over a sliding period of 31 days. The rainiest month in Gharb is November, with an average rainfall of 65 mm. The dry period of the year lasts 4 months, from May 21st to September 20th. The least rainy month in Gharb is July, with an average rainfall of 1 mm (Figure 2).

In the Loukkos region, the rainy period of the year lasts 8.4 months, from September 14th to May 27th, with rainfall of at least 13 mm over a sliding period of 31 days. The rainiest month in Loukkos is November, with an average rainfall of 94 mm. The dry period of the year lasts 3.6 months, from May 27th to September 14th. The least rainy month in Loukkos is July, with an average rainfall of 1 mm.

#### 3.3.2. Emberger bioclimatic quotient

The Emberger bioclimatic quotient (Q_2_) was calculated according to the three formulas mentioned above (Table 1). Table 8 represents the *Q*_2_ values calculated in the two regions Gharb and Loukkos.

**Table 8:** Emberger bioclimatic quotient (Q_2_) of the two regions Gharb and Loukkos.

	Gharb	Loukkos
Q_2_ Mokhtari et al. (2013)	102.91	171.70
Q_2_ Stewart (1968)	103.15	171.50
Q_2_ Emberger (1942)	102.89	171.69
Means	102.98	171.63

**Figure 3:**
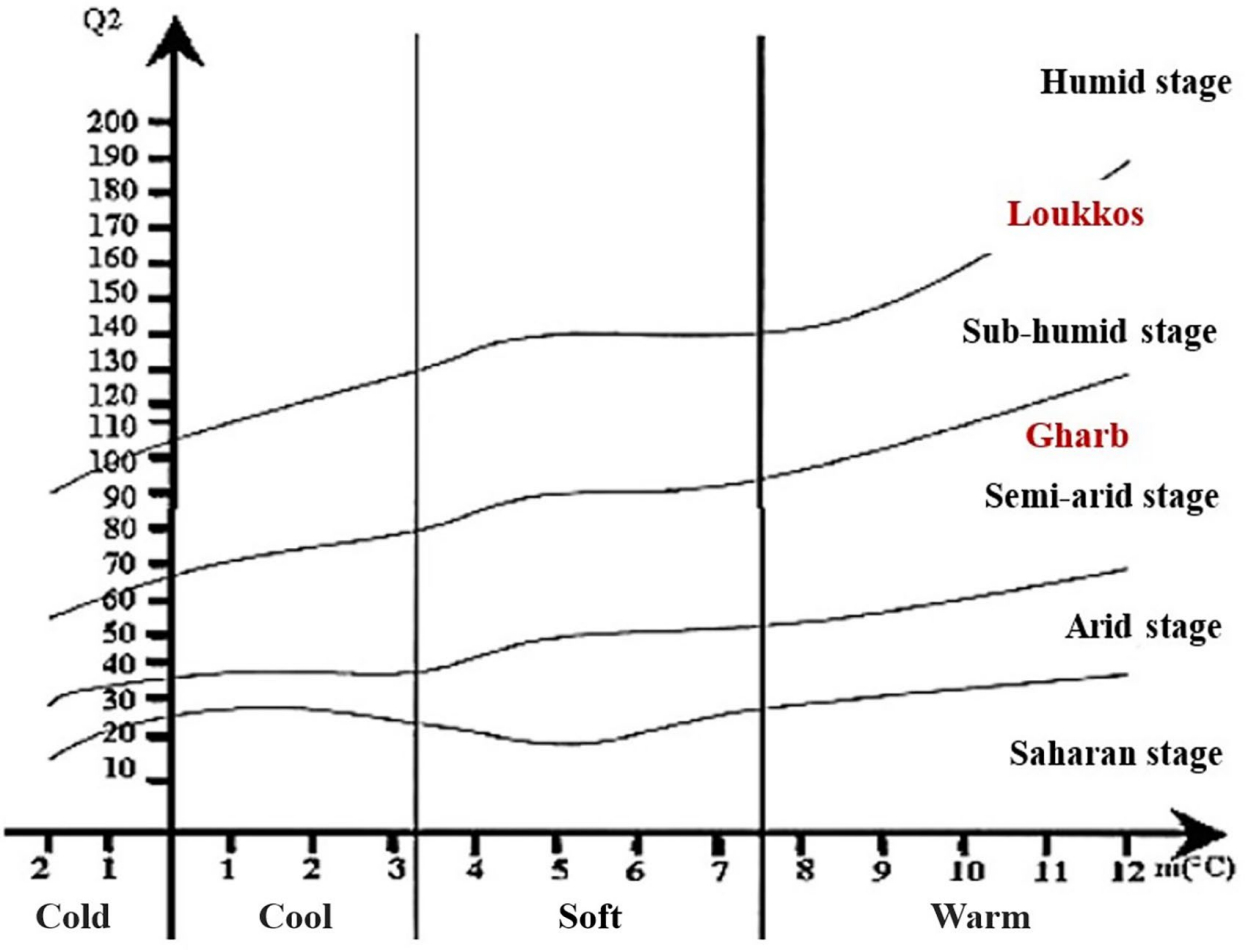
Emberger diagram of the bioclimatic stages of the two regions Gharb and Loukkos.

The climatic parameters allowed us to locate the two target regions Gharb and Loukkos on the Emberger diagram (Figure 3). According to the latter, the Gharb region (Rabat-Sale-Kenitra) is located on the semi-arid stage. While, the Loukkos (Tangier-Tetouan-Al Hoceima) is located on the sub-humid stage. In Gharb, the climate is clearly Mediterranean in the inland cities and Mediterranean with an oceanic influence in the coastal cities. In Loukkos, the climate is Mediterranean in the coasts and surroundings, rather continental and with periods of heavy snowfall in the inland cities of the region. Bioclimatic indices have a strong influence on plant growth as well as its contamination, which explains the differential effect on the prevalence of bacteria in strawberry samples from Gharb (Tables 2, 3) and on the prevalence of fungi in strawberry samples from Loukkos (Tables 4, 5).

Strawberries are perishable fruits that, during handling, can be attacked by certain microorganisms, especially bacteria. Also, the main causes of strawberry diseases during storage and shelf life are the development of rots caused by a series of fungi. Climatic conditions were suspected to have a differential effect on the prevalence of bacteria in the Gharb strawberry samples and on the prevalence of fungi in the Loukkos strawberry samples. A climatological study was necessary to explain this regional distribution.

Analysis of the association between microbial indicators and climatology demonstrates the plausibility of the idea that climatology can affect foodborne pathogens pre-harvest. Climatic conditions are an important factor to consider regarding microbial contamination of fresh products (Machado-Moreira et al., 2019). It is known that changes in climatic conditions determine the development of strawberry plants, affecting flowering, fruiting, and fruit quality, among other characteristics (Khammayom et al., 2022). However, it is pertinent to mention that the influence of climatic conditions on plant contamination may be specific to each microorganism/plant/climatic conditions combination, meaning that no clear trend can be established (Ward et al., 2015). Climatic conditions during the flowering and ripening of strawberry fruit influence fruit quality and microbial contamination as well as affect shelf life more than the irrigation method used (Parikka et al., 2009).

Climatic conditions, including precipitation, humidity, temperature, wind, and orchard management activities, have previously been shown to be correlated with the risk of post-harvest fungal disease development during apple storage (Dutot et al., 2013; Bui et al., 2021). Studies on plant pathogens have shown that high humidity and moderate temperatures promote fungal growth (Griffin, 1996; Rowan et al., 1999). *B. cinerea* infections are correlated positively with relative humidity and negatively with temperature (Feliziani and Romanazzi, 2016). Weather factors, particularly humidity and temperature, had a direct influence on fungal abundance and richness in the air and on vegetation leaf surfaces, showing large differences among habitats (Talley et al., 2002).

According to the bacteriological and mycological study, there is a difference in the contamination level of strawberry fruits according to the locality of origin. The Gharb region shows a prevalence of bacterial contamination and the Loukkos region reveals a prevalence of fungal contamination. The climatological analyses carried out confirm this hypothesis. Evidently, post-harvest management determines food quality and safety, market competitiveness, and producer profits. Temperature and humidity management is the most important factor to control in order to minimize spoilage of strawberry fruit and maximize its post-harvest life. This management must take into consideration the climatic conditions of the cultivated areas.

## 4. Conclusion

Pre-harvest weather conditions affect strawberries’ quality and susceptibility to post-harvest pathogen attacks. The objective of this survey was to study the microbiological quality and the prevalence of major foodborne pathogens on strawberry fruits in the Gharb and Loukkos regions of Morocco. Thus, to establish a correlation between the post-harvest microbial load and the pre-harvest bioclimatic conditions. The Gharb region has a prevalence of fungal contamination, while the Loukkos region has a prevalence of bacterial contamination. The climatological study confirms this hypothesis. Post-harvest storage and conditioning of strawberries must take into consideration the climatology of the growing regions to improve food safety practices for a good strawberry agricultural system.

## Data availability statement

The original contributions presented in the study are included in the article/supplementary material, further inquiries can be directed to the corresponding author.

## Author contributions

All authors listed have made a substantial, direct, and intellectual contribution to the work, and approved it for publication.

## Funding

This work was supported by the PRIMA project (Enhancing Mediterranean Fresh Produce Shelf-Life using Sustainable Preservative Technologies and Communicating Knowledge on Dynamic Shelf-Life using Food Cloud Services and Predictive Modeling BIOFRESHCLOUD).

## Conflict of interest

The authors declare that the research was conducted in the absence of any commercial or financial relationships that could be construed as a potential conflict of interest.

## Publisher’s note

All claims expressed in this article are solely those of the authors and do not necessarily represent those of their affiliated organizations, or those of the publisher, the editors and the reviewers. Any product that may be evaluated in this article, or claim that may be made by its manufacturer, is not guaranteed or endorsed by the publisher.
